# Low diagnostic yield of repeat urine cultures in hospitalized patients at a tertiary center in Northern California, 2023–2024

**DOI:** 10.1017/ice.2025.10248

**Published:** 2025-09

**Authors:** Eugenia Miranti, Mindy M. Sampson, John Shepard, Guillermo Rodriguez-Nava, Karen McIntyre, Erika Paola Viana Cardenas, Barbara W. Trautner, Jorge L. Salinas

**Affiliations:** 1 Division of Infectious Diseases, Stanford University Hospital, Stanford, CA, USA; 2 Michael E. DeBakey Veterans Affairs Medical Center and Section of Health Services Research, Baylor College of Medicine, Houston, TX, USA

## Abstract

We analyzed the diagnostic yield of repeat urine cultures in a retrospective study of adult inpatients. Most urine cultures repeated at less than 6 days provided redundant information. This was true whether the index culture was positive or negative, and whether the threshold for positivity was 10,000 or 100,000 CFU/mL.

## Introduction

Urine cultures are frequently ordered in hospitalized patients, though inappropriate ordering practices remain a major problem.^1^ The reduction of inappropriate duplicate diagnostic testing is an important facet of diagnostic stewardship and reducing health care costs.^2^ Prior work has demonstrated that short interval (less than 48 hour) repeat urine cultures detect new bacteriuria at rates of less than 5%.^3,4^ Less is known about the value of repeating urine cultures at longer time intervals. We evaluated the diagnostic yield of repeating a positive or negative urine culture at different intervals among hospitalized patients.

## Methods

We performed a retrospective study at Stanford Health Care, a 650-bed teaching hospital. We included all hospitalized patients ≥18 years of age who had at least one urine culture performed during their hospitalization during January 2023—February 2024. We included urine cultures collected in patients with or without urinary catheters. We excluded urine cultures collected from nephrostomies/urostomies, from ileal loop/conduits, and samples aspirated directly from the kidney in the operating room.

A culture was labeled as an “index” culture if it was followed by a subsequent (repeat) urine culture. Bacteriuria was defined as growth of a bacterial species in quantities >100,000 colony forming units per milliliter (CFU/mL). Urine cultures that grew candida alone were classified as “negative.” Polymicrobial urine cultures were included in the analysis if they grew at least one bacterial species at quantities >100,000 CFU/mL.

We defined the diagnostic yield as the percent of repeat urine cultures with new bacteriuria with a species not detected in the index culture. We performed sensitivity analyses for the threshold of bacterial growth (>100,000 CFU/mL and >10,000 CFU/mL) and for the time elapsed from index to repeat culture: 0–3; 3–6; and 6–9 days.

We report descriptive results using frequencies and percents. Bivariate comparisons were made using Wilcoxon rank sum and the chi-square tests. Analyses were performed using STATA version 18 (College Station, TX). The study was approved by the Institutional Review Board of Stanford University.

## Results

A total of 6,955 urine cultures were obtained from 6,091 patients. There were 663 patients who had repeat urine cultures collected, with numbers of repeats ranging from 1–8, for a total of 864 index-repeat pairs. In total, 12% of all urine cultures were repeats. Most bacterial species detected were gram-negative rods (67%). The most detected species were *E. coli* (39%), *E. faecalis* (13%), and *K. pneumoniae* (13%).

A higher proportion of patients who had repeat urine cultures were male, had negative index cultures, died in the hospital, and had their initial urine culture collected in inpatient wards; their median duration of hospitalization was longer, with more intensive care unit days (Table 1).

**Table 1.** Comparison of patients with and without repeat urine cultures among 6091 hospitalized patients at Stanford Health Care during January 2023–February 2024

Demographics	Patients without repeat urine cultures (*N* = 5428)	Patients with repeat urine cultures (*N* = 663)	*P*-value
Age (years) ^a^	68.5 (54, 80)	66 (52, 76)	< 0.001
Sex (*N*, % Female)	3,422 (63%)	370 (56%)	< 0.001
Race (*N*, % White)	2,681 (49%)	334 (50%)	0.63
Ethnicity (*N*, % Latino)	1,254 (23%)	146 (22%)	0.53
Hospitalization data			
Admission length (days)^a^	5 (3, 10)	16 (7, 29)	< 0.001
Intensive care days^a^	0 (0, 0)	0 (0, 2)	< 0.001
Death during encounter (*N*, %)	177 (3%)	53 (8%)	< 0.001
Location of initial culture collection (*N*, %)^b^			
Emergency department	3,059 (56%)	264 (40%)	< 0.001
Observation unit	209 (4%)	21 (3%)	0.38
Intensive care unit	267 (5%)	55 (8%)	< 0.001
Medical ward	1,775 (33%)	312 (47%)	< 0.001
Operating room	90 (2%)	8 (1%)	0.38
Psychiatric unit	28 (<1%)	3 (<1%)	0.83
Source of initial culture (*N*, %)			
Clean catch/straight catheterization	4,674 (86%)	514 (78%)	< 0.001
Indwelling catheter	754 (14%)	149 (22%)	< 0.001
Initial culture data (*N*, %)			
Bacteriuria >100k CFU/mL^c^	1,656 (31%)	172 (26%)	0.015
Bacteriuria <100k CFU/mL ^c^	3,772 (69%)	491 (71%)	0.015
Bacteriuria >10k CFU/mL^c^	2,733 (50%)	311 (47%)	0.377
Bacteriuria <10k CFU/mL^c^	2,697 (50%)	352 (53%)	0.377

a Median (Interquartile Range)

b Percents may not sum to 100% due to rounding

c Colony Forming Units per milliliter.

Repeat urine cultures were usually collected from the same unit as the index culture (58%) and from the same source as the index culture (81%); that is, both via clean catch/straight catheterization or both from an indwelling catheter. The median time to repeat urine culture was 4 days (range: 0–78 days).

Of the 864 index cultures, 340 (39%) were repeated at 0–3 days (241 negative index, 99 positive index), 192 at 3–6 days (157 negative, 35 positive), and 110 (13%) at 6–9 days (87 negative, 23 positive). The remaining 222 (26%) urine cultures were repeated at >9 days (159 negative, 63 positive).

When negative index cultures were repeated within 0–3 days, the diagnostic yield was only 9% (21/241). Diagnostic yield at 3–6 days was 10% (16/157), not significantly higher compared to 0–3 days (*p* = 0.620). Diagnostic yield at 6–9 days was 18% (16/87); a significant increase compared to the 0–3 days group (*p* = 0.014). When positive index cultures were repeated at 0–3 days, the diagnostic yield was 5% (5/99). Diagnostic yield at 3–6 days was 9% (3/35), not a significant difference (*p* = 0.45). Yield significantly increased to 17% (4/23) at 6–9 days (*p* = 0.041). Presence of a urinary catheter at the time of collection of the index specimen was not a risk factor for new bacteriuria in the repeat culture (11% in those without catheter had a new bacterial species detected in the repeat culture, vs. 13% in those with a catheter, *p* = 0.475), nor was presence of a urinary catheter at the time of repeat urine culture collection (12% of those without catheter vs. 10% in those with catheter, *p* = 0.332).

**Figure 1.**
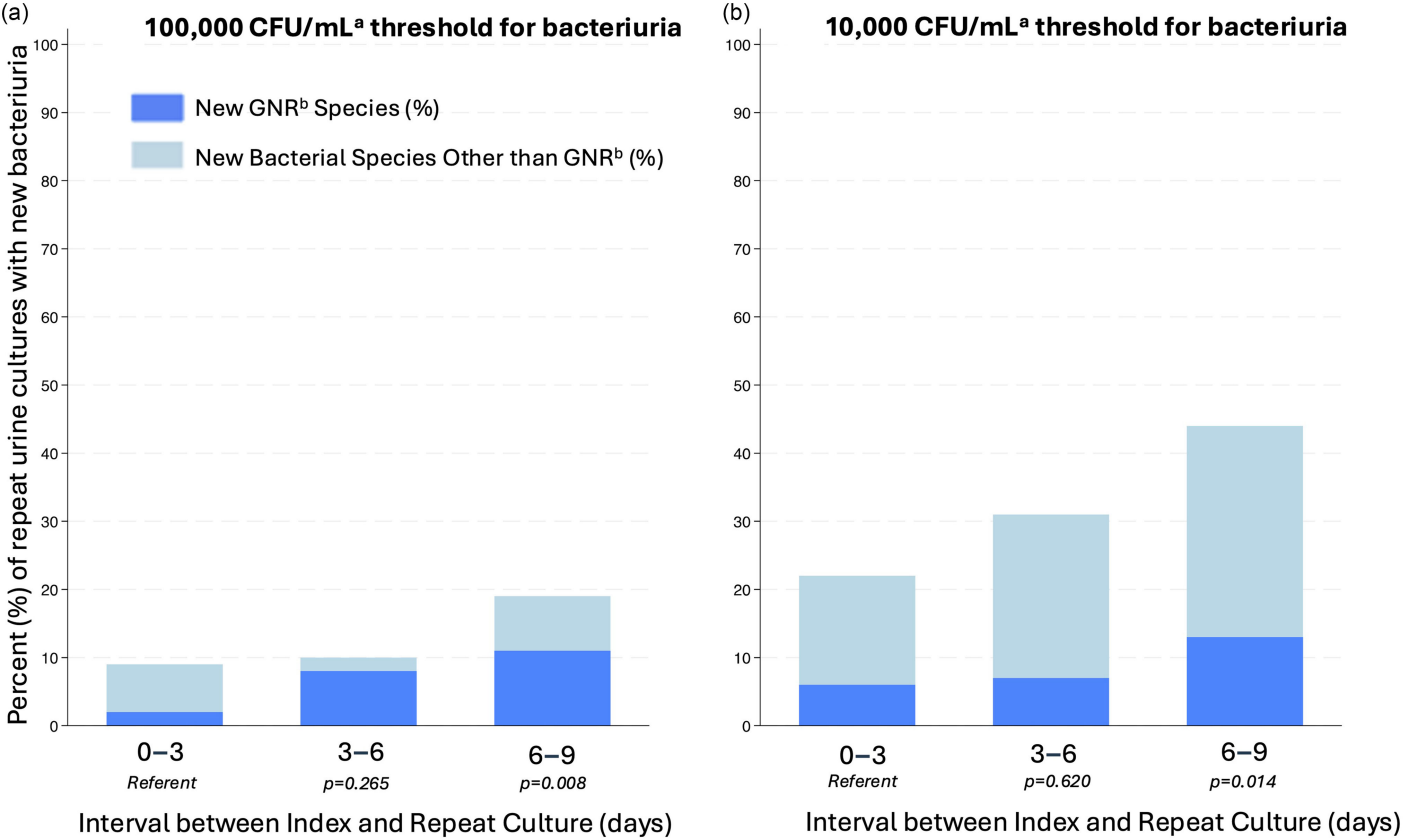
Diagnostic yield of repeating a negative urine culture, stratified by threshold for significant bacteriuria among hospitalized patients at Stanford Health Care during January 2023–February 2024 ^a^Colony Forming Units per milliliter ^b^Gram-Negative Rod.

When the threshold for positivity was adjusted to 10,000 CFU/mL, more bacteriuria was detected overall, but primarily of gram-positive organisms. The rate of detection of new gram-negative bacteriuria remained low (<10%) until 6 or more days after index culture (Figure 1).

## Discussion

We found that most urine cultures repeated within 6 days of the index culture have low diagnostic yield. We found repeat urine cultures were low yield, even with conservative thresholds for new bacteriuria (10,000 CFU/mL), and even when extending the repeat period up to six days. These findings were also true regardless of the result of the index culture. Diagnostic excellence is an important aspect of quality of medical care, and the Centers for Disease Control and Prevention have recently published a roadmap for its implementation.^5^ Out of all infectious disease tests, urine cultures are among the top candidates for reduction.

We found that patients who have repeat urine cultures likely represent a sicker population than those who do not, given their longer hospitalizations with more intensive care days and higher proportion who died in hospital. This is in line with prior work demonstrating highest diagnostic intensity in critical care patients, often with limited diagnostic yield.^6^ Providers may feel compelled to send more tests in patients who are not clinically improving or who have been in the hospital for longer.

It has previously been observed that duplicate orders are common when patients are transferred from the emergency department to inpatient units.^3,7^ However, in our study population, we found that a lower proportion of patients with repeat urine cultures had an initial urine culture collected in the emergency department. Perhaps this is because patients who had urine cultures repeated did not present to the hospital with urinary complaints. We speculate that patients who have repeat urine cultures may have more undifferentiated presentations, motivating providers to order more diagnostic tests, including more urine cultures.

This study has several limitations. It was conducted in an academic medical center, so generalizability to nonteaching, community hospitals may be limited, as these may have different ordering practices. Its retrospective nature creates susceptibility to sampling bias. We lack data for repeat urine cultures collected from patients following their hospitalization; a repeat urine culture collected at an outside institution would not be captured. Bacteriuria is an imperfect surrogate for urinary tract infection. Chart review was not conducted, so presence of urinary symptoms and receipt of antibiotics are unknown.

These limitations notwithstanding, we present robust sensitivity analyses accounting for bacteriuria thresholds, time from index to repeat culture, and the result of the index culture. In conclusion, repeat urine cultures add little new clinical data and thus repeat urine cultures are an excellent target for diagnostic stewardship efforts.
